# The Endodontic Space

**DOI:** 10.3390/healthcare11040628

**Published:** 2023-02-20

**Authors:** Alfredo Iandolo

**Affiliations:** Department of Medicine, Surgery and Dentistry “Scuola Medica Salernitana”, University of Salerno, Via S. Allende, 84081 Baronissi, Italy; aiandolo@unisa.it

Modern endodontics allows for the use of new materials and techniques in treating and saving teeth in a simple and reproducible way.

Importantly, before discussing new materials and protocols, it is essential to highlight the influence of the endodontic space on our treatment success. Moreover, it is misperceived to consider the root canal system as a simple area because, in fact, it is very complex when analyzed microscopically.

In addition to being composed of the main canal, the endodontic space consists of different macro- and micro-lateral anatomies, such as lateral canals, isthmuses, loops, branches, apical deltas, multiple exit ports, and dentinal tubules. Consequently, manual or rotating files cannot reach and clean these microscopic spaces. For this reason, it is essential to understand the complexity of the endodontic space and implement dedicated protocols for their cleaning [1,2,3].

Success in endodontics is achieved by eliminating or decreasing the bacterial load within the whole root canal system. Therefore, as mentioned above, shaping alone is ineffective in obtaining this result. Hence, it is crucial to employ the correct irrigants and activation techniques, using high-performance techniques in such a way as to reach and act in these lateral micro-spaces.

Generally, there are different irrigant activation techniques: sonic, ultrasonic, laser, heat, and similar. Indeed, only after accurately shaping and cleaning the endodontic space is it possible to proceed with the obturation phase [4,5,6].

Currently, the latest generation of sealers has been introduced to improve the quality of the filling phase, together with gutta-percha cones [7,8]. These sealers offer excellent advantages, such as high biocompatibility, a high pH, faster healing, micro-expansion inside the canal, and much more [9,10,11].

In conclusion, with the help of modern technologies and new materials, the success of our therapies will be enhanced; however, we must keep in mind the complexity of endodontic anatomy, because only after studying it well can we implement our protocols.

This Special Issue aims to focus on the most modern materials and techniques. Moreover, it aims to discuss the advances in operative microscopes, CBCT, modern access cavities, conservative shaping, active cleaning, obturation with the latest generation of sealers, and surgical endodontics.
